# Gelatin as a Hæmostatic

**Published:** 1901-02

**Authors:** 


### GELATIN AS A HÆMOSTATIC.

Schlesinger gives a long account of the introduction and use of gelatin as a hæmostatic (*Centralbl. f. Grenz. d. Med. u. Chir.*, 1900, Bd. iii. 209). In 1896 Dastre and Floresco found that intravenous injection of a gelatin solution in animals caused the blood to coagulate more rapidly when withdrawn from the vessels. Control experiments proved that this is not due to the well-known "setting" properties of gelatin. They also found that it antagonized the power of peptone in hindering coagulation of the blood. Camus and Gley explained the result by attributing it to the acidity of the gelatin solution; but Floresco, while admitting that the acid reaction has a certain influence, showed that neutralized gelatin is also active. Bauermeister explained it by an action in the leucocytes which are killed by the gelatin, and in dying produce the ferment which brings about blood coagulation. When injected subcutaneously the same effect is observed, but there is still much uncertainty regarding the rate and method of its absorption.

Carnot treated a severe case of epistaxis in a hæmophile by the injection of a five per cent. gelatin solution into the nostril, and the bleeding stopped almost at once. A second similar case was successfully treated by means of a ten per cent. solution. Many other forms of external hemorrhages can be controlled in the same way or by means of tampons soaked in the solution. Carnot says the gelatin must be carefully sterilized, which is best accomplished by heating it to 100° C. on two successive days. The flask can then be plugged, and on steeping it in warm water its contents become fluid, and are ready to use. For local application he uses a five to ten per cent. solution in a 0.7 per cent. salt solution. Sirdey has also used it locally in metrorrhagia, bleeding from piles, epistaxis; and successful cases have been reported by others. Its action is rapid and lasting, and without danger. Poliakow reports a case of hemorrhage from gastric ulcer, in which six ounces of a ten per cent. solution given thrice in twenty-four hours stopped the bleeding. Bleeding from the lower bowel can be controlled by injections. Hæmoptysis does not seem to be much benefited by its internal administration.

Nogues has treated cases of vesical bleeding by injections of gelatin solution into the bladder. The viscus is first thoroughly washed out with boric lotion, and then several small injections of a five per cent. gelatin solution are given; the bladder is again washed out, and is ultimately left partially filled with it.

The subcutaneous injection of gelatin to stop hemorrhage was first used by Lancereaux and Paulesco. Cases of hæmoptysis, hæmophilic bleedings, and bleeding from toxæmia can all be treated in this way. Several physicians have used with success a two per cent. solution in hæmoptysis, in bleeding from the bowel in typhoid, and in hæmophilia. As a rule, about six ounces is injected daily for several days in succession. In obstinate hemorrhage after various operations it is also effective, and it has controlled in several cases the bleeding of purpura hemorrhagica.

In cholemic bleedings Kehr states that three or four injections suffice to control the tendency to hemorrhage. His conclusions are: (1) A solution of gelatin increases the coagulability of healthy and pathological blood. (2) This occurs after local application and subcutaneous administrations. (3) It is harmless if antiseptic precautions are carefully carried out. (4) It can be used in all cases of bleeding from the most various causes. (5) Its value as a prophylactic before operation can only be established after further experience. (6) For subcutaneous injection a one or two per cent. solution is best; for local application, a five to ten per cent. solution at a temperature of 98° to 100° F. (7) It must be sterilized. (8) Heart and kidney disease contraindicate its use.—*Edinburgh Medical Journal,* July, 1900.

